# Modeling Product Manufacturing Reliability with Quality Variations Centered on the Multilayered Coupling Operational Characteristics of Intelligent Manufacturing Systems

**DOI:** 10.3390/s20195677

**Published:** 2020-10-05

**Authors:** Anqi Zhang, Yihai He, Xiao Han, Yao Li, Xiuzhen Yang, Zixuan Zhang

**Affiliations:** School of Reliability and Systems Engineering, Beihang University, Beijing 100083, China; zhanganqibuaa@buaa.edu.cn (A.Z.); buaahanxiao@buaa.edu.cn (X.H.); 41601409@xs.ustb.edu.cn (Y.L.); 20171843@stu.neu.edu.cn (X.Y.); dzb@mail.xaufe.edu.cn (Z.Z.)

**Keywords:** product manufacturing reliability, operational characteristics, PQR chain, coupling effect, integrated reliability modeling

## Abstract

For intelligent manufacturing systems, there are many deviations in operational characteristics, and the coupling effect of harmful operational characteristics leads to the variations in quality of the work-in-process (WIP) and the degradation of the reliability of the finished product, which is reflected as a loss of product manufacturing reliability. However, few studies on the modeling of product manufacturing reliability and mechanism analysis consider the operating mechanism and the coupling of characteristics. Thus, a novel modeling approach based on quality variations centered on the coupling of operational characteristics is proposed to analyze the formation mechanism of product manufacturing reliability. First, the PQR chain containing the co-effects among the manufacturing system performance (P), the manufacturing process quality (Q), and the product manufacturing reliability (R) is elaborated. The connotation of product manufacturing reliability is defined, multilayered operational characteristics are determined, and operational data are collected by smart sensors. Second, on the basis of the coupling effect in the PQR chain, a multilayered product quality variation model is proposed by mining operational characteristic data obtained from sensors. Third, an integrated product manufacturing reliability model is presented on the basis of the variation propagation mechanism of the multilayered product quality variation model. Finally, a camshaft manufacturing reliability analysis is conducted to verify the validity of the proposed method. The method proposed in this paper proved to be effective for evaluating and predicting the product reliability in the smart manufacturing process.

## 1. Introduction

In intelligent manufacturing systems, with the widespread application of intelligent technology and advanced sensors, traditional resources are converted into intelligent data and information [1]. Intelligent manufacturing systems, which involve men, machines, materials, methods, environments, and measurements, are complicated. With the operation of processing, these factors are coupled to affect the quality of a produced product [2]. As the output of the manufacturing process, the quality of Work In Process (WIP) is directly related to the operational status of the manufacturing system [3]. The quality variations of WIP are transferred between processes, leading to the degradation of the reliability of the finished product. To fundamentally improve and pre-evaluate the reliability of the product, clarifying the operational mechanism of characteristics in the manufacturing system and its effect on the finished product is a predictive means.

During the operation of the manufacturing system, the dynamically changing machine status and the uncertain manufacturing environment interact with each other to cause performance degradation of the manufacturing system, which is the direct cause of the degradation of WIP quality. Han et al. [4] evaluated the health status of a manufacturing system by using the operating data collected by smart sensors, including the production machine, task, and product. Given that the machine is the basic unit of a manufacturing process, Sun et al. [5] established a model of machine reliability by considering the interaction between tool degradation and product quality. Chen et al. [6] evaluated the state of a machine by using its reliability, and then built a production scheduling model that incorporates machine reliability to help decision makers determine the optimal scheduling strategy. In modern manufacturing systems, low-cost sensors are widely deployed, which have led to data-driven machine health monitoring methods gaining the attention of the manufacturing industry. Zhao et al. [7] proposed a deep learning model, which is not limited to specific types of machines; thus, it is widely used to monitor and evaluate the health status of machines. The new capsule network model based on convolutional neural networks is proposed, which is used to diagnose bearing faults and evaluate the health status of rotating machines [8]. Ou et al. [9] monitored and predicted the tool wear status of CNC machine tools by using a stacked sparse auto-encoder feature learning method. In addition, Levitin et al. [10] considered the impact of the random replacement time of spare components on the 1-out-of-N system, and then proposed a numerical algorithm for the instantaneous availability of the multi-state standby system, the expected availability within the system task time. Lu et al. [11] monitored the status of quality-related components in the system to assess the quality loss and system failure rate of the final product and proposed a state-based multistate manufacturing system maintenance strategy. Real-time data collection based on sensors is an effective way to implement manufacturing process monitoring [12]. Syafrudin [13] developed a big data processing platform that can collect massive and real-time data from sensors during the manufacturing process. Rani et al. [14] developed a monitoring machine condition method to diagnose machine faults in real time, which uses compressed signal processing to improve detection efficiency. Li et al. [15] collected the data that affects the manufacturing system performance through intelligent sensors connected to the cloud, including state data of equipment and parts, so as to make the operation decision of the manufacturing system. However, in the actual production process, various characteristics are coupled during operation [16,17]; the analysis of a single factor is not conducive to comprehensively assessing the performance of the manufacturing system and cannot accurately predict product quality. In addition, the real-time monitoring of the production process cannot easily estimate product quality in advance. Therefore, in order to comprehensively evaluate the performance of the manufacturing system, it is necessary to consider the coupling mechanism of the characteristics on the basis of the operational data of the manufacturing system collected by smart sensors [18].

Given that the variation propagation of harmful characteristics in the manufacturing system can lead to the degradation of product quality, the Quality–Reliability co-effect model is used to construct the interaction relationship between manufacturing process quality and output product reliability [19]. Lu et al. [20] determined the variables in the manufacturing process that affect product quality and quantitatively described the effects of process variables on product quality on the basis of the response model. Zhou et al. [21] described the interaction mechanism of product quality and station reliability; in view of this feature, a preventive maintenance strategy of the manufacturing system is proposed. Considering the interaction between product quality and machine performance in the manufacturing system, Ye et al. [22] proposed an integrated reliability evaluation model of the manufacturing system subsequently. In addition, Stream of Variation (SoV) theory describes that in the multi-station manufacturing system, the variation of the process is transmitted to cause the variation of the output product size. SoV is widely used in multistage complex manufacturing systems to control and improve product quality [23,24]. Abella’n-Nebot et al. [25] proposed an extended SoV model on the basis of process tolerances, which clarify the effects of variables in the machining process on the costs of the manufacturing system and product quality. Zhang et al. [26] constructed a state space model to characterize the relationship between the source variation and the final assembly variation considering the part manufacturing error, fixture position error, and relocation-induced error. Given that the quality variation of a product is propagated among stations, Du et al. [27] used the state transition probability of key parameters in the manufacturing process to estimate the quality state of the final product by using novel Markov models. These studies focused on expressing the co-effect between product size variation and process variable in the manufacturing system, thereby providing a solid theoretical foundation for investigating the coupling effect of operational characteristics on the quality of WIP. However, only a small number of studies have been conducted on the interaction between product reliability after the manufacturing process and quality variation in the product size.

Inspired by the evolution of reliability during the product life cycle, production reliability directly determines the product quality level that enters the hands of users [28]. Therefore, evaluating product reliability after the manufacturing process is valuable for studying whether a product has reached the reliability goal before in the market. Before a product is delivered to a customer, the reliability acceptance test can verify whether product reliability remains within the prescribed level during the mass production stage. Given that traditional RAT is time-consuming and inefficient, Tsai et al. [29] selected the key quality characteristics of a product for testing and determined its acceptance probability. Wang et al. [30] improved the accelerated stress reliability acceptance test plan by optimizing the decision variables in the test. However, these studies ignore the interference of harmful factors in the manufacturing process and cannot fundamentally guide the reliability improvement program of a product. From another perspective, due to defects in the manufacturing process, hidden defects in the product only explode with time and stress in the initial stage of use [31]. He et al. believed that the high failure rate of a product in the early stages of use could be considered a manifestation of low production reliability [32,33]. However, these studies focused on expressing the reliability of the product under the interference of the initial environmental stress, and could not accurately express product reliability after the manufacturing process.

Considering that variations in the operational characteristics in the manufacturing system are continuously transmitted and coupled during the machining process, the quality of WIP is eventually reduced, and the reliability of the finished product after the manufacturing process is degraded. Therefore, a product manufacturing reliability model based on multilayered product quality variations affected by the coupling of operational characteristics is proposed. Compared with previous research, the main contributions of this paper are as follows:

(1) The connotation of product manufacturing reliability based on the PQR chain is expounded, and the three-level multilayered operational characteristics in the manufacturing system that affect product quality are determined.
(2) Multilayered product quality variation models considering the coupling effect of operational characteristics, which include machine operation status, processing fluctuation, and WIP quality degradation, are established.
(3) An integrated product manufacturing reliability model is established, considering the variation propagation of product quality on the basis of the multilayered product quality variation models.

The remainder of this paper is organized as follows: Section 2 discusses the basic theory of product manufacturing reliability and types of operational characteristics. Section 3 presents the model construction of the multilayered product quality variation models, considering the coupling effect of multilevel operational characteristics. Section 4 establishes the product manufacturing reliability model on the basis of the multilayered product quality variation model. Section 5 introduces the camshaft manufacturing process to verify the effectiveness of the proposed method. Section 6 provides the conclusion.

## 2. Basics of Product Manufacturing Reliability Modeling

### 2.1. Formation Connotation of Product Manufacturing Reliability Based on the PQR Chain

**Figure 1:**
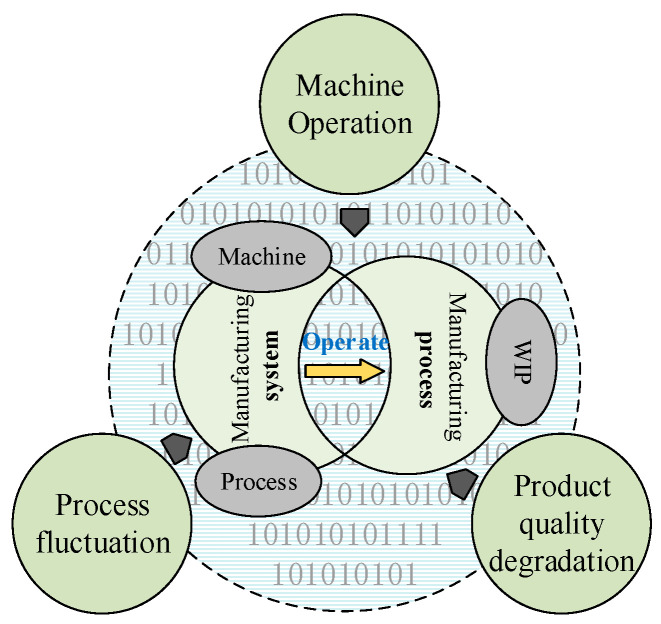
Composition and operation of the manufacturing system.

During the product life cycle, product reliability gradually deteriorates over time. Specifically, the manufacturing stage is a critical period in which a product is finished. As previously mentioned, the propagation of harmful characteristics in the manufacturing system can lead to variations of product quality, that is, the performance status of the manufacturing system determines the quality of the finished product. Intelligent manufacturing systems involve machine, process, and production environments, as well as humans; with the operation of the system, materials are converted into WIP, and then assembled to form a finished product in a dynamic manufacturing process. To evaluate the performance status of the manufacturing system, machine, machining process, and WIP are included in this modeling (Figure 1).

As shown in Figure 1, the operation of characteristics within the manufacturing system, such as the machine, process, and WIP, affects the quality level of WIP, which is considered the output of the manufacturing process. During the operation of the manufacturing system, the machine is disturbed by adverse factors in the production environment, and the operational state of the machine is degraded. With this process, the quality variation of the degraded machine is transferred, and an abnormal fluctuation in the machining process occurs. As the output of the manufacturing process, the quality of WIP degenerates with the abnormal fluctuation in the process.

On the basis of the characteristics of the manufacturing system, the conceptual model is established, considering the relationship among manufacturing system performance (P), manufacturing process quality (Q), and product manufacturing reliability (R).

**Figure 2:**
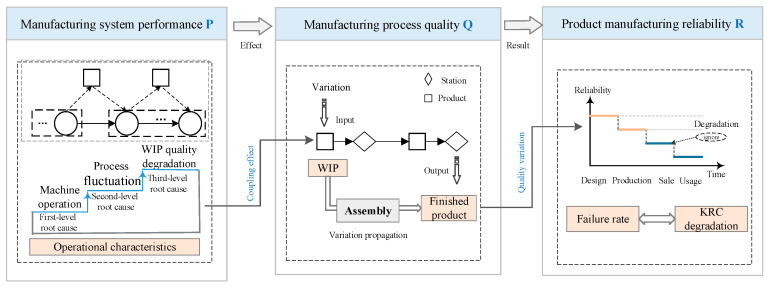
Conceptual modeling of product manufacturing reliability.

As illustrated in Figure 2, the performance of the manufacturing system (P) depends on the state of the multilayered operational characteristics. As the manufacturing system runs, the dynamic operational characteristics of the manufacturing system, including the multiple machine operation, the fluctuation level of each process, and the quality degradation of WIP, have an interactive effect in turn. The uncertain production environment inevitably leads to an irreversible degradation trend of the system characteristic state and system performance state from the ideal state to complete failure. The quality of the manufacturing process (Q) is influenced by those of the WIP and the finished product after assembly, which are threatened by the transmission and coupling of variations in multilayered operational characteristics, that is, the PQ effect of manufacturing system performance on manufacturing process quality. After the manufacturing process, the product is finally finished. Product manufacturing reliability (R) is the critical stage for achieving lossless transfer from the design phase to the use phase. However, due to the propagation of variations in the manufacturing process, the reliability goal of the design undergoes loss during the production stage that is usually manifested by the degradation of the critical reliability characteristics (CRCs) of the product, or in other words, increased failure rate. This is the QR effect of the manufacturing process quality on the product manufacturing reliability. Based on this, the product manufacturing reliability is defined as the probability that CRCs meet the design reliability requirements. The formation of product manufacturing reliability depends in the performance of the manufacturing system and the quality of the manufacturing process based on PQR chain.

Based on the reverse PQ and QR effects, the coupling effect of the three-level operational characteristics is the root cause of the degradation of product manufacturing reliability. Therefore, clarifying the mechanism of product manufacturing reliability degradation on the basis of operational characteristics data is an effective means to guarantee product quality.

### 2.2. Conceptual Model of Product Manufacturing Reliability Concerning Multilayered Operational Characteristics

**Figure 3:**
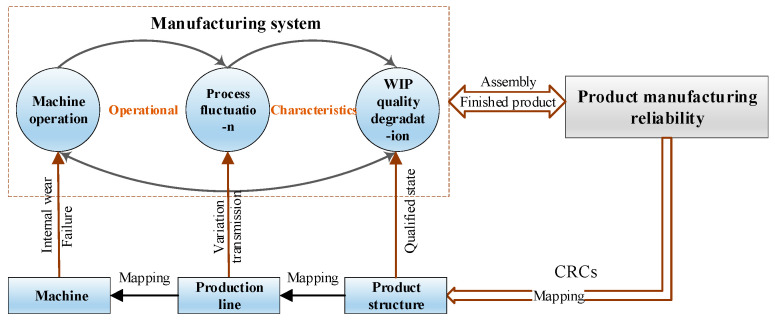
Multilayered model of operational characteristics.

As mentioned in the PQR chain, the coupling of operational characteristics gradually degrades the performance of the manufacturing system. Subsequently, WIP with variations enters the assembly process, and the reliability of the finished product is reduced due to the transmission of variations. Therefore, clarifying the mechanism of operational characteristics is a prerequisite for product manufacturing reliability modeling. A multilayered model of operational characteristics is established, as displayed in Figure 3.

Figure 3 shows the formation mechanism of product manufacturing reliability that focuses on the interaction of operational characteristics in the manufacturing system. Assume a product has m CRCs, that is, C={c1,c2,⋯cm}. The opposite events of the failure rates of CRCs can characterize product manufacturing reliability to a certain extent.

(1) $$R_{m}=\bar{\lambda }\left(c1,c2,\cdots ,cm\right)$$

Subsequently, the CRCs are mapped to the product structure domain. In this process, the finished product is broken down into *w* related WTP; as the quality variation of WIP is transferred during the assembly process, the reliability of the finished product degenerates. Therefore, Equation (2) is expressed as follows:

(2) $$Rm=\bar{V}\left(Q_{1},Q_{2},\cdots Q_{w}\right)$$

Once WIP is determined, the production line and the related machines are mapped and determined in sequence according to the production regulations. In the manufacturing system, the machine operation, process fluctuation, and WIP quality affect one another and thus ultimately leads to continuous WIP quality degradation. During the operation of the manufacturing system, the machine is affected by internal degradation and random failure. Therefore, the operational state of the machine is continuously degraded. Multiple machines form a process, which fluctuates abnormally, and the stability is threatened due to machine variation. Furthermore, the quality of WIP output by the process is degraded, and the transfer of the defective WIP between the processes cause the machine to degrade, such as tool wear. Therefore, Equation (3) is established.

(3) $$Q_{w}\left(t\right)=F_{H,D}\left[\left(H,o\right),\left(D,f\right)\right]$$

where $F_{H,D}$ is the integrated distribution of WIP quality degradation, considering machine state distribution H(t) and process fluctuation distribution D(t).

Combining Equations (1)–(3), the estimated model of product manufacturing reliability is expressed as Equation (4).

(4) $$Rm=F_{Q,H,D}\left[\bar{V}\left(Q_{1∼w}\right),\left(H,o\right),\left(D,f\right)\right]$$

### 2.3. Multilayered Operational Characteristics Data Obtained from Smart Sensors

Modern manufacturing systems are intelligent and highly automated, and various fine materials, complex machine, advanced technology and data analysis methods run through the entire manufacturing process. With the operation of multilayered characteristics in the manufacturing system, the data containing the performance status information of the manufacturing system is generated, and there is a certain degree of correlation and coupling between these operational data. The acquisition of operational characteristic data is the basis for modeling the variation transmission in the manufacturing system, and is the primary condition for realizing the product manufacturing reliability analysis. It is worth noting that the widespread application of information technology makes it possible to collect all-round characteristic status data. Among them, particularly importantly, the wide deployment of smart sensors in the production line enables dynamic machine status and product quality information to be monitored and acquired in real time. In this study, the state data of the multilayered operational characteristics is collected by smart sensors.

**Figure 4:**
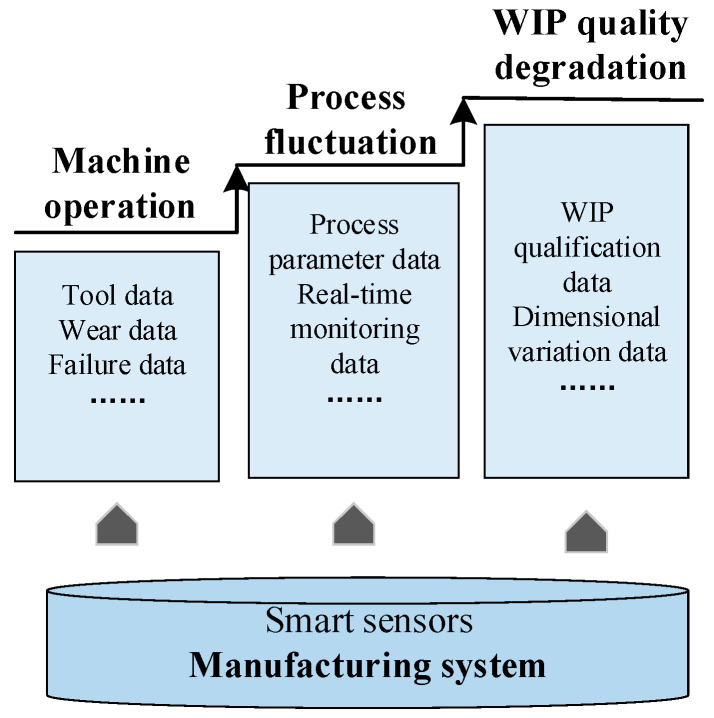
Collection of operational characteristics data from sensors.

As mentioned in Section 2.2, the multilayered operational characteristics data in the manufacturing system are the basis for estimating product manufacturing reliability. The deployment of various types of sensors in the intelligent manufacturing system realizes the real-time collection of characteristics operational data, including machine operation, process fluctuation, and WIP quality. Sufficient operational characteristic data are the basis for analyzing the coupling effect and provide a solid data foundation for determining the formation mechanism of product manufacturing reliability. Various types of operational characteristic data are collected by sensors in the manufacturing system (Figure 4). 

As shown in Figure 4, operational characteristic data are collected by various types of sensors in the manufacturing system. As the system runs, the operational state of production machines continuously degrades, which is reflected in the real-time data of the wear of specific parts of the machines and failure data captured by sensors. Such as, the index data of the clamp tightening pressure is sensed by the smart pressure sensor. The intelligent sound sensor judges the noise signal during the operation of the machine. Subsequently, relevant process parameters are determined. The variation of each process is monitored and collected by sensors in real time. The related displacement sensor can accurately monitor the trajectory of the tool displacement. As the output of the process, the quality variation of WIP is detected and recorded. For example, the dimension and surface roughness of the WIP are quickly detected by smart sensors.

After the operational characteristics data are collected, the multilayered quality variation models are successively established, considering the coupling effect mechanism of the operational characteristics in Section 3.

## 3. Multilayered Quality Variation Modeling Focused on Coupling Operational Characteristics

### 3.1. Degradation of Machine Operation State Partially Affected by WIP Quality

During the operation of the machine, its operation state is in an irreversible degradation, which is affected by WIP quality, internal wear, and random failure, as illustrated in Figure 3. In general, according to the form of machine failure, it can be divided into wear-out failure and sudden failure. It is assumed that wear-out failure and sudden failure are independent of each other.

First, on the one hand, WIP is transferred to the next station machine after one process on the basis of the coupled multilayered model of operational characteristics. When WIP size deviates, the machine tool wears out. The discrete defective WIP shocks the machine state and causes the machine operation state to degenerate. Assuming that the shock frequency n(t) of WIP in the time range from 0 to *t* follows a non-homogeneous Poisson distribution, that is,

(5) $$n\left(t\right)=\int_{0}^{t}\lambda_{P}\left(x\right)dx$$

where $\lambda_{P}\left(x\right)$ is the jump intensity function, which is determined through the sensors connected to the cloud on the device in the actual production. 

The discrete wear amount gradually accumulates without loss of generality, and the machine wear caused by WIP variation $\left\{D_{P}\left(t\right),t>0\right\}$ follows the Wiener process.

(6) $$D_{P}\left(t\right)=D_{P}\left(n\left(t\right)\right)=D_{P}\left(n\right)=\theta n+\sigma D_{B}\left(n\right)$$

where θ is the drift coefficient, and σ is the diffusion coefficient; both are determined by the fitting dynamic wear data obtained from smart sensors. $D_{B}\left(n\right)$ is the standard Wiener process. The shock of WIP is independent of each other, and the degradation increment meets the following conditions.

(7) $$D_{P}\left(0\right)=0,$$

(8) $$\Delta D_{Pi}=D_{Pi}-D_{P\left(i-1\right)}.$$

The degradation increment is only related to the shock frequency and follows a normal distribution.

(9) $$\Delta D_{Pi}∼N\left(\theta \Delta n_{i},\sigma^{2}\Delta n_{i}\right)$$

On the other hand, another cause of machine wear is that the machine gradually fatigues under stress loads, and the degradation of the machine operation state due to internal fatigue wear $\left\{D_{I}\left(t\right),t>0\right\}$ follows the Gamma distribution with shape parameter α and scale parameter β.

(10) $$D_{I}\left(t\right)∼Gamma\left(\beta ,\alpha \right)$$

Its probability density function is shown below.

(11) $$d\left(t\right)=\frac{\beta^{\alpha }}{\Gamma \left(\alpha \right)}t^{\alpha -1}e^{-\beta t}$$

The wear degradation process of the machine consists of the cumulative wear of WIP shock and internal fatigue wear. When the accumulated degradation reaches $D_{th}$, the machine fails.

(12) $$D_{P,I}\left(t\right)=D_{P}\left(t\right)+D_{I}\left(t\right)$$

Therefore, the probability that the amount of machine degradation does not reach the failure threshold is:

(13) $$\begin{array}{cc} P_{m}\left(t\right) & =P\left(D_{P,I}\left(t\right)<D_{th}\right)\\ & =P\left(D_{P}\left(t\right)+D_{I}\left(t\right)<D_{th}\right) \end{array}$$

Secondly, the sudden shock also has a fatal effect on the machine operation state. The frequency of sudden shock follows a non-homogeneous Poisson distribution process.

(14) $$m\left(t\right)=\int_{0}^{t}\lambda_{S}\left(x\right)dx.$$

Notably, the sudden shock is assumed to cause the machine to fail directly with probability p. On the contrary, it has no effect on the machine with probability 1−p. The probability that the sudden shock does not cause the machine to fail is:

(15) $$P_{T}\left(t\right)=pm\left(t\right)$$

Finally, the reliability of the machine operation state without failure is:

(16) $$P_{e}=P_{m}\left(t\right)P_{T}\left(t\right)$$

### 3.2. Fluctuation of Machining Process Based on State Entropy

The production process is a combination of machines and operators under a specific technology and can change the quality characteristics of WIP. During the operation of the manufacturing system, the fluctuation of the process determines whether the process can continuously produce qualified WIP. That is, the stability of the process is an important factor in the quality of the manufactured product.

At present, the quality of the process is controlled and analyzed by the control chart, but post- and intra-event analyses cannot effectively predict the fluctuation of the process. Therefore, by synthesizing the influencing factors of process fluctuations, predicting the process fluctuation state is meaningful to optimize the system. 

Given that the machine is the smallest unit that constitutes the production process, machine errors flow into the process and cause it to deviate. Previous process errors also flow into the next process. Therefore, the composite variation propagation model of process i is constructed as Equation (17).

(17) $$y_{i}=\sigma_{i}^{T}y_{i-1,g}+D_{P,I}\left(t\right)a_{i}+\varepsilon$$

where $y_{i}=\sum_{g=1}^{t}\left|\bar{y_{i,g}}-y_{m,g}\right|$ represents the sum of the deviation monitored by sensors between the actual size and standard size of the *m* dimensional characteristics in process *i*, and $\sigma_{i}^{T}$ is a vector set of transfer coefficients from process *i−1* to process *i*. $D_{P,I}\left(t\right)={\left[D_{P,I,1},D_{P,I,2},\cdots D_{P,I,n}\right]}^{T}$ is the set of machine operational state parameters involved in process *i* and follows the distribution of Equation (12). $a_{i}$ is the conversion coefficient from machine to process, ε is the random error, and the mean is 0.

Subsequently, let

(18) $$Y={\left[\begin{array}{cccc} y_{i,1} & y_{i,2} & \cdots & y_{i,n} \end{array}\right]}^{T}$$

(19) $$X={\left[\begin{array}{cccc} \sigma_{i,1} & \sigma_{i,2} & \cdots & \sigma_{i,t} \end{array}\right]}^{T}$$

(20) $$A={\left[\begin{array}{cccc} y_{i-1,1,1} & y_{i-1,1,2} & \cdots & y_{i-1,1,t}\\ y_{i-1,2,1} & y_{i-1,2,2} & \cdots & y_{i-1,2,t}\\ \vdots & \vdots & \cdots & \vdots \\ y_{i-1,n,1} & y_{i-1,n,2} & \cdots & y_{i-1,n,t} \end{array}\right]}_{n\times t}$$

(21) $$S=D_{P,I}\left(t\right)a_{i}$$

(22) $$\nu ={\left[\begin{array}{cccc} \varepsilon_{1} & \varepsilon_{2} & \cdots & \varepsilon_{n} \end{array}\right]}^{T}$$

Therefore, the process variation propagation model (Equation (17)) is transformed into:

(23) $$\underset{n\times 1}{Y}=\underset{n\times t}{A}\underset{t\times 1}{X}+\underset{n\times 1}{S}+\underset{n\times 1}{\nu }$$

Equation (23) is a standard semiparametric regression model. Transfer coefficients $\sigma_{i}^{T}$ and conversion coefficient $a_{i}$ are estimated by the fitting of historical production data on the basis of R language. With the machine operational state and the previous process status, the current process status is determined. Evidently, process fluctuations automatically turn to a chaotic state as the variation propagates. The entropy of the process varies dynamically with the degree of fluctuation. In this study, state entropy is used to characterize the degree of process fluctuation (Equation (24)).

(24) $$H\left(y_{i}\right)=-p_{i}\log p_{i}$$

where $p_{i}=\frac{y_{i}+y_{m}}{y_{m}}$ is the ratio of the actual size to the expected size; when $p_{i}$ is 1, the entropy value is 0, which is the ideal state.

Finally, the fluctuation state of the process is expressed as follows:

(25) H(y)=[H(y1),H(y2)⋯]

### 3.3. WIP Quality Depends on the Process Fluctuation and the Machine State

In the dynamic manufacturing process, WIP quality is usually affected by process fluctuation and machine operational state, as illustrated in Figure 3. With the degradation of the machine operational state and the disorder increase of the process fluctuation, the qualification rate of WIP degenerates. Noise factors originating from raw materials and human factors in the production environment can also threaten WIP quality. The quality level of WIP can be characterized by the variation of key dimension characteristic parameters. Therefore, Equation (26) is established.

(26) $$q_{i}\left(t\right)=\varphi_{i}+v_{i}H{\left(t\right)}^{T}+b_{i}D{\left(t\right)}^{T}+H\left(t\right)a_{i}D{\left(t\right)}^{T}$$

where $v_{i}$ and $b_{i}$ represent the influence coefficient vector of process fluctuation H(t) and machine operational state D(t), respectively. $a_{i}$ is the interaction matrix between process H(t) and machine D(t). $\varphi_{i}$ is the vector noise factor in the production environment.

After the process fluctuation and machine operational state are determined, the dimension characteristics of WIP are reasonably predicted and obtained. In fact, the qualification rate of such dimension characteristics is unrelated to the number of products produced. Therefore, modeling the qualification rate of WIP on the basis of limited prediction samples is necessary. Assume that the qualification rate of i-th WIP is $Q_{i}$, and the probability of failure is $1-Q_{i}$. $Q_{i}$ is a uniformly distributed sample from [0, 1]. The probability of $Q_{i}$ must be calculated. The conditional probability of $Q_{i}$ follows the *Beta* distribution (Equation (27)), where the value of $Q_{i}$ is independent of the number of samples.

(27) $$Q_{i}∼Beta_{1}\left(a,b\right)$$

where *a* and *b* are the parameters of the Beta distribution.

On the basis of the above prior information, moving the probability distribution curve and updating the parameters of the Beta distribution to model the new distribution results are completed. After the prediction of response model Equation (26), *s* samples are assumed to exist among which x qualified WIP are found. Given *s* samples and *x* qualified conditions, $Q_{i}$ is subject to

(28) $$Q_{i}∼Beta_{2}\left(a+x,b+\left(s-x\right)\right)$$

Furthermore, the expectation of the Beta distribution is used to express the value of $Q_{i}$, and the qualification rate of i-th WIP is expressed as follows:

(29) $$Q_{i}=\frac{a+x}{a+b+s}$$

With the passage of time, the operational state of the machine gradually degrades, and the resulting processes tend to be chaotic. Therefore, the qualification rate is a series of discrete degraded values:

(30) Q(t)=[Q1,Q2,Q3,⋯]

## 4. Integrated Modeling Approach of Product Manufacturing Reliability

### 4.1. Framework of the Product Manufacturing Reliability Modeling 

**Figure 5:**
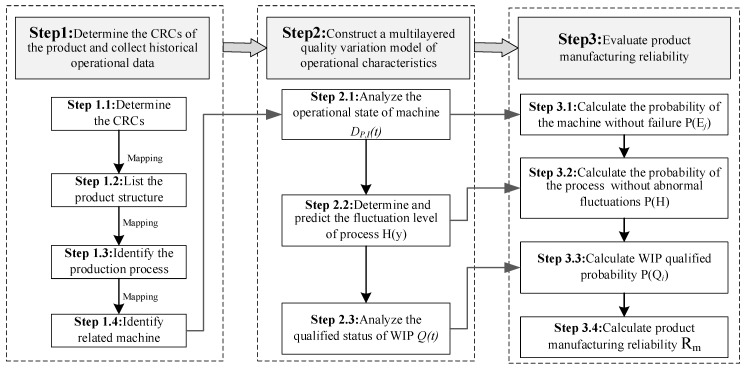
Workflow of the product manufacturing reliability modeling.

Based on the coupling variation model of operational characteristics, the construction framework of the integrated product manufacturing reliability model is illustrated in Figure 5.

As shown in Figure 5, the historical operational data of related structures, processes, and machines are sequentially mapped and collected on the basis of the known CRCs of the product. Subsequently, the multilayered variation model of operational characteristics with machine, process, and WIP is established separately. Finally, the reliability of the operational characteristics is evaluated, which enables the evaluation and modeling of product manufacturing reliability.

**Step 1:** Determine the CRCs of the product and collect data on historical operational characteristics. Once the CRCs are determined, the relevant WIP that has undergone the assembly process is identified according to the product structure. Related production processes and machines are identified through systematic mapping and decomposition according to production regulations. At the same time, relevant historical operational data are collected.

**Step 2:** Construct a multilayered quality variation model of operational characteristics. According to the collected historical operational data of the machine, the wear amount of the machine itself, the shock number of WIP with variations, and the shock number of random failures are determined. The distribution of the machine operational state is fitted, and the distribution parameters are obtained in Section 3.1. On the basis of the historical operational machine state and the WIP size characteristics, the coefficients in the process variation transfer model are determined. The process fluctuation state is also predicted on the basis of the machine operational distribution results. Combining historical machines, process state, and WIP quality, the response variable model is clear. Therefore, WIP quality is presumed on the basis of machine operation distributions and process fluctuations at arbitrary periods.

**Step 3:** Model the product manufacturing reliability. The probability that the machine will not fail at time *t* is estimated on the basis of the machine’s operational state distribution. When the probability that the machine without failure at time *t* is known, the probability of the corresponding process without abnormal fluctuation before time *t* is estimated. Similarly, when the probability that the machine without failure and the process without abnormal fluctuation at time *t* are known, the WIP qualified rate is evaluated. Finally, the finished product manufacturing reliability at time *t* is estimated.

### 4.2. Product Manufacturing Reliability Model Integrates the Quality Variations of Multilayered Operational Characteristics

In the workflow of product manufacturing reliability modeling, product mapping and the multilayered quality variation model of operational characteristics are completed on the basis of the historical operational data collected by smart sensors. In the conceptual model of product manufacturing reliability, WIP qualification is directly related to product manufacturing reliability. As previously mentioned, by determining the probability of the machine without failure and the probability of the process without abnormal fluctuation, the probability that WIP qualification meets the manufacturing requirements is determined. Furthermore, the reliability of a product composed of multiple WIPs after the manufacturing process is determined.

First, the probability that $P\left(E_{rj}\right)$ corresponds to the j-th machine in the r-th process that runs until *t* does not fail is expressed as follows:

(31) $$P\left(E_{rj}\right)=P_{e}=P_{m}\left(t\right)P_{T}\left(t\right)$$

Second, according to the fluctuation model of the process established in Step 2, when process H_r_ runs to time *t*, the fluctuation distribution of process H_r_ is H(y)=[H(y1),H(y2)⋯], and the fluctuation value satisfies H(yi)>H(ym) when abnormal fluctuations occur. Therefore, $P\left(H_{r}\right)$ represents the probability that no abnormal fluctuation occurs at the time *t* of the running process H_r_. 

For convenience, assume that process H_r_ is composed of J machines connected in series. When the probability of the machine without failure involved in the process is known, the probability of the corresponding process without abnormal fluctuation is updated as follows:

(32) $$\tilde{P}\left(H_{r}\right)=P\left(H_{r}\right)\times \prod_{j=1}^{J}P\left(E_{rj}\right)$$

Third, when the dimensional variation of WIP exceeds the production requirements, WIP is judged as unqualified. According to the WIP variation model established on the basis of Equation (26), the qualification trend of the i-th WIP over running time *t* is expressed as $P\left(Q_{i}\right)$ on the basis of Equation (30). Assume that the i-th WIP consists of M series processes. When the probability of the machine without failure involved in WIP is known, the qualification trend of WIP is updated on the basis of Equations (32) and (33).

(33) $$\tilde{P}\left(Q_{i}\right)=P\left(Q_{i}\right)\times \prod_{r=1}^{M}\prod_{j=1}^{J}\tilde{P}\left(H_{r}\right)P\left(E_{rj}\right)$$

Finally, on the basis of Equation (2), the finished product manufacturing reliability composed of m WIP is expressed as follows:

(34) $$R_{m}=\prod_{i=1}^{w}\tilde{P}\left(Q_{i}\right).$$

## 5. Case Study

### 5.1. Background

**Figure 6:**
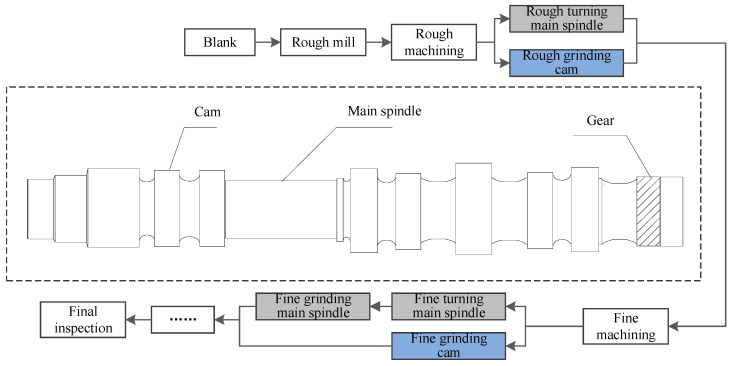
Camshaft structure diagram and manufacturing process.

The cylinder head is the basic component used by the engine to close the cylinder. Among them, as the core component of the cylinder head, the camshaft can drive the opening and closing of the valve. The camshaft is a typical shaft part, and its machining process is complicated and difficult. Moreover, the machining process level of the camshaft directly affects the power performance of the engine. The processing of the camshaft is completed in the manufacturing system, as displayed in Figure 6.

As shown in the structure diagram, the coaxiality, surface roughness, processing size, and other characteristics of the camshaft have high requirements. Correspondingly, as the core component of the camshaft, the machining processes of the main spindle and cam are complicated. Referring to the production process regulations, the manufacturing process of the main spindle is composed of rough turning spindle, fine turning spindle, and fine grinding spindle, involving four machines. Meanwhile, cam processing experiences rough grinding cam and fine grinding cam, involving two machines. 

### 5.2. Numerical Example

This part takes the manufacturing process of the engine cylinder head camshaft as the research object. According to the workflow of product manufacturing reliability modeling in Figure 5, the manufacturing reliability of the camshaft is obtained, which verifies the effectiveness of the method proposed in this paper. The detailed steps are shown below. 

**Step 1:** Historical data information collection and mapping stage. The concentricity, surface roughness, and size of the camshaft are considered CRCs. According to the structural design principle of the camshaft, the main spindle and cam are determined as the WIP of the camshaft. According to the manufacturing process, related processes and machines are mapped and obtained, which are presented in Table 1. Subsequently, the historical fault data and dynamic wear data of the machine collected and stored by the sensors are extracted. At the same time, the characteristics variation of the WIP is also recorded, such as WIP dimension, surface roughness, etc.

**Step 2:** Construction stage of the multilayered quality variation model of operational characteristics. The operational state of the machine, the fluctuation state of the process, and the qualified state of WIP are respectively determined according to the fitting of the historical operational data obtained from sensors in Step 1. The parameters of machines M1.1.1–M2.2.1 are determined according to the method described in Section 3.2. The fitting parameter results of each machine are shown in Table 2. 

Subsequently, the data information of processes P1.1–P2.2 is collected. The transfer and conversion coefficients are fitted. The variation propagation expression of each process is shown below. On this basis, the fluctuation of each process can be obtained.

$$Y_{P1.1}\left(t\right)=0.525\times D_{P,I1.1}\left(t\right)+{\left(0.1941,-0.0146,\cdots \right)}_{n\times 1}^{T},$$

$$Y_{P1.2}\left(t\right)=A_{1.2}\left(t\right)\left(\begin{array}{c} 0.0034\\ 0.1027 \end{array}\right)+D_{P,I1.2}\left(t\right)\left(\begin{array}{c} 0.0016\\ 0.039 \end{array}\right)+{\left(0.0046,-0.0257,\cdots \right)}_{n\times 1}^{T},$$

$$Y_{P1.3}\left(t\right)=A_{1.3}\left(t\right)\left(\begin{array}{c} 0.6012\\ 0.3048 \end{array}\right)+0.013D_{P,I1.3}\left(t\right)+{\left(0.0012,-0.0142,\cdots \right)}_{n\times 1}^{T},$$

$$Y_{P2.1}\left(t\right)=0.696\times D_{P,I2.1}\left(t\right)+{\left(-0.0026,0.0288,\cdots \right)}_{n\times 1}^{T},$$

$$Y_{P2.2}\left(t\right)=A_{2.2}\left(t\right)\left(\begin{array}{c} 0.0681\\ 0.1532 \end{array}\right)+0.085\times D_{P,I2.2}\left(t\right)+{\left(0.0026,0.0182,\cdots \right)}_{n\times 1}^{T}.$$

**Figure 7:**
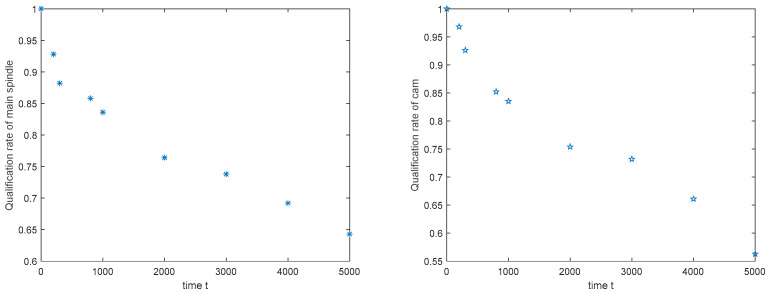
WIP qualification rate over time.

Next, the quality variation model parameters of the main spindle and cam are estimated as presented below. On this basis, the qualification rate of each WIP is obtained according to Equations (27)–(29). The change trends of the qualification rates of the main spindle and cam are analyzed, and the results are displayed in Figure 7.

$$v_{1}={\left(\begin{array}{ccc} 0.113 & 0.152 & 0.236 \end{array}\right)}_{n\times 3}$$

$$v_{2}={\left(\begin{array}{cc} 0.096 & 0.146 \end{array}\right)}_{n\times 2},$$

$$b_{1}={\left(\begin{array}{cccc} 0.075 & 0.162 & 0.154 & 0.203 \end{array}\right)}_{n\times 4}$$

$$b_{2}={\left(\begin{array}{cc} 0.243 & 0.175 \end{array}\right)}_{n\times 2},$$

$$a_{1}=\left(\begin{array}{cccc} 0.225 & 0 & 0 & 0\\ 0 & 0.0016 & 0.009 & 0\\ 0 & 0 & 0 & 0.013 \end{array}\right)$$

$$a_{2}=\left(\begin{array}{cc} 0.196 & 0\\ 0 & 0.085 \end{array}\right).$$

**Figure 8:**
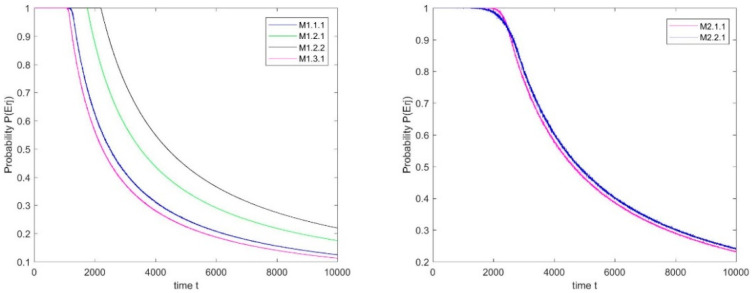
Probability of machines without failure of main spindle and cam.

**Step3:** Camshaft manufacturing reliability modeling stage. According to the machine parameters in Table 2, the probability curves of each machine without failure over time during operation are shown in Figure 8. 

**Figure 9:**
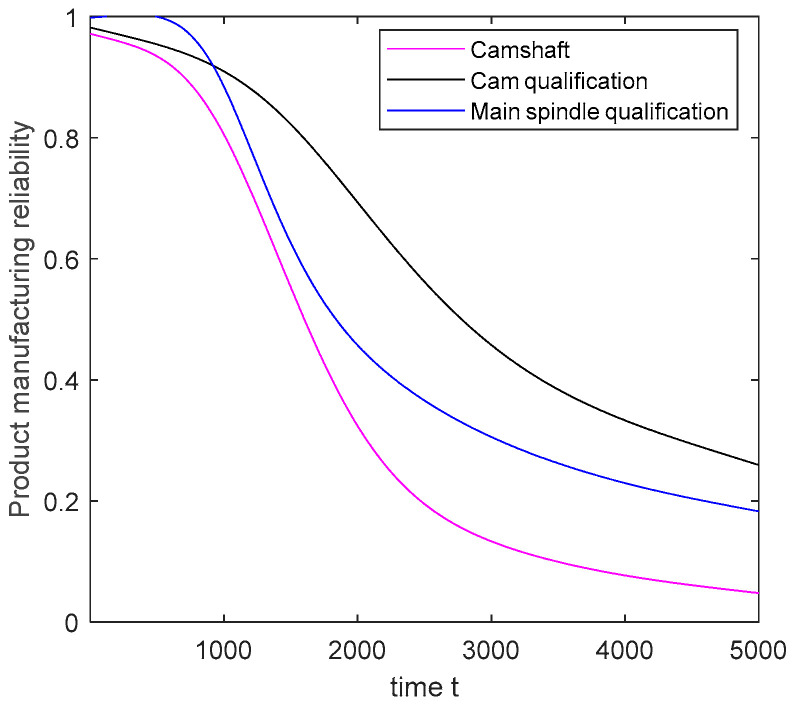
Camshaft manufacturing reliability and product qualification.

Notably, after the probability of machines without failure over time is acquired, the probability of the processes being without abnormal fluctuation over time can be estimated using Equation (32). Subsequently, the qualification trends of the main spindle and cam are expressed in Figure 9. Finally, during the manufacturing process of the camshaft, the camshaft manufacturing reliability is determined using Equation (34) (Figure 9). 

### 5.3. Results and Discussion

#### 5.3.1. Result Analysis

In Section 5.2, through the fitting and prediction of the operation data of the machine, process, and WIP collected by sensors during the camshaft manufacturing process, the degradation process of the machine, the fluctuation state of the process, and the qualification of WIP were described in turn. The manufacturing reliability of the camshaft was presumed to be based on the normal levels of the machines, processes and WIP that provide a reference for controlling the degradation of product manufacturing reliability in advance.

#### 5.3.2. Comparative Study

A comparative study with the traditional variation transfer model is conducted, thus verifying the advancement of the product manufacturing reliability modeling mentioned in this paper. 

**Figure 10:**
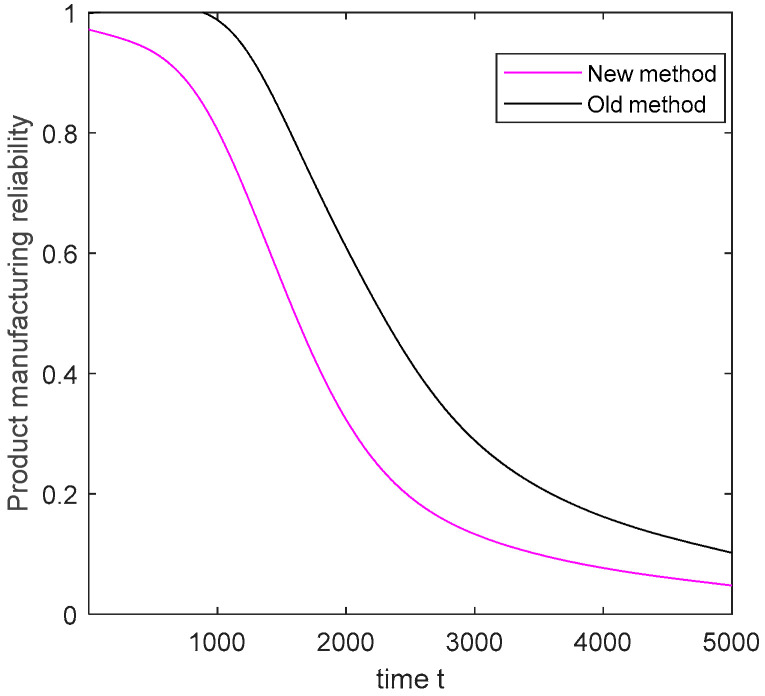
Comparative analysis of camshaft manufacturing reliability.

The traditional variation transfer model ignores the coupling effect of the characteristics in the manufacturing system, including the interaction between the machine and WIP and the influence of the machine operation state on process fluctuation. The degradation mechanisms of the machine operation state and the process fluctuation state are ignored. Therefore, the degradation process of the machine and process are inaccurately expressed. The real-time data of the machines and processes collected by sensors are used to estimate the parameters of the model. Subsequently, product reliability is estimated. As shown in Figure 10, the camshaft manufacturing reliability over time is estimated using the traditional variation transfer model and the method proposed in this study, respectively.

As illustrated in Figure 10, the camshaft manufacturing reliability under the two methods is estimated. When the coupling effect of operational characteristics in the intelligent manufacturing system is not considered, camshaft manufacturing reliability is overestimated. In the manufacturing system, product manufacturing reliability overestimation leads to an increase in the actual product failure rate, and the machine cannot be repaired in time. Evidently, the method proposed in this paper is effective and advanced. The results show that fully considering the coupling effect of operational characteristics and the degradation mechanisms of the machine, process, and WIP improves the accuracy of product manufacturing reliability to a certain extent, which can reduce product losses and provide a reference for subsequent machine maintenance plans.

## 6. Conclusions

Due to the transmission and coupling of multiple operational variations in complex manufacturing systems, the reliability of the finished product is lower than the design target. After the manufacturing process, the product directly enters into use. Therefore, estimating product manufacturing reliability is significant. The variations of the operational characteristics in the manufacturing system are coupled with each other, resulting in a reduction in the manufacturing reliability of the finished product. Therefore, evaluating the manufacturing reliability of the finished product by considering only a single characteristic variable is inaccurate. This study focuses on the coupling effect of the multilayered operational characteristics in the intelligent manufacturing system and proposes the modeling of product manufacturing reliability. Fortunately, in the current intelligent manufacturing system, compared with traditional sensors, smart sensors can diagnose and calibrate the state information of the machine and products through internal integrated circuits and network technology to store state data in the database, realizing the real-time monitoring of the manufacturing process. Abundant operating data provides a solid foundation for the analysis of coupling relationships and the prediction of the finished product reliability. On this basis, this article completes the modeling of product manufacturing reliability based on the following aspects. First, considering the interaction among the manufacturing system, the manufacturing process, and the finished product, the PQR chain is proposed, and the connotation and formation mechanisms of product manufacturing reliability are clarified. At this time, the coupling effect of operational characteristics in the system is considered to be the primary factor that affects product manufacturing reliability. To ensure an accurate analysis of the coupling mechanism, operational data is collected by smart sensors. Second, the coupling effect of multilayered operational characteristics is further analyzed. On this basis, the quality variation model of each operational characteristic is established. Third, product manufacturing reliability is estimated by integrating the probability of machine operation without failure, the probability of process without abnormal fluctuation, and the qualification rate of WIP. Finally, the method proposed in this study is verified by the manufacturing process of the camshaft on the cylinder head. The results indicate that camshaft manufacturing reliability gradually degrades with system operation. Future studies will carry out in-depth research with respect to the following aspects:

1. The production task will be considered one of the operational characteristics in the manufacturing system.
2. Interactions among production machines will be further considered.

## Figures and Tables

**Table 1:** Camshaft manufacturing processes and machines.

WIP	Process	Machine	Process	Machine
Main spindle	P1.1 Rough turning	M1.1.1 CNC lathe	P1.2 Fine turning	M1.2.1 CNC lathe
				M1.2.2 CNC lathe
			P1.3 Fine grinding	M1.3.1CNC grinder
Cam	P2.1 Rough grinding	M2.1.1Cam grinder	P2.2 Fine grinding	M2.2.1Cam grinder

**Table 2:** Parameters of each machine operational state.

	M1.1.1	M1.2.1	M1.2.2	M1.3.1	M2.1.1	M2.2.1
n(t)	0.6t	0.8t	0.5t	0.12t	0.08t	0.6t
θ(10^−4^)	2.8	3.2	2.8	3.5	1.2	2.5
σ(10^−3^)	12	1.2	1.8	1.2	2.5	80
α	4.42	5.62	4.62	5.02	5.45	4.12
β(10^−4^)	2.5	3.2	2.7	3.8	1.4	60
Threshold	0.22	0.45	0.31	0.05	0.025	0.45
P_T_(t)	0.9996	0.9996	0.9997	0.9998	0.9997	0.9998
